# Supplementary material for: Effectiveness and Safety of Ayurvedic Medicines in Type 2 Diabetes Mellitus Management: A Systematic Review and Meta-Analysis
Source: Front Pharmacol. 2022 Jun 8;13:821810. doi: 10.3389/fphar.2022.821810 (PMC9213670; doi:10.3389/fphar.2022.821810)
Supplement: Supplementary file 1 [file DataSheet1.zip › Supplementary Material/Forest and Funnel Plots/Gynostemma pentaphyllum/Fasting insulin.pdf]

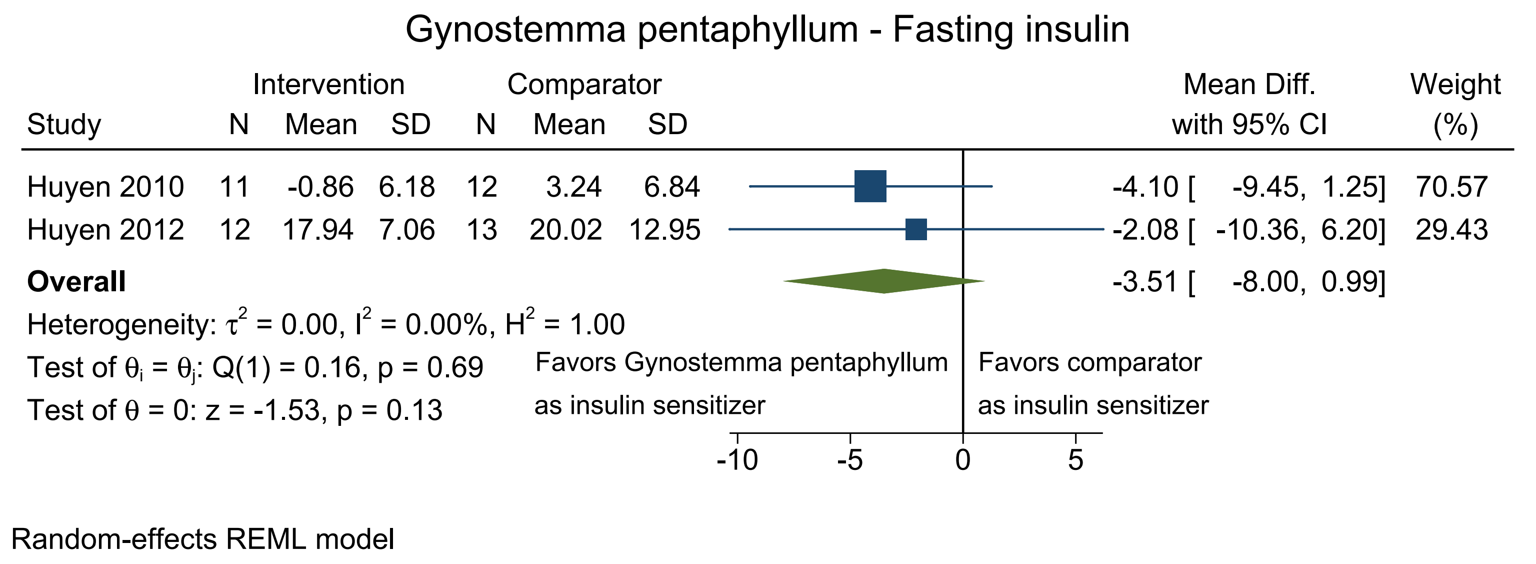
Gynostemma pentaphyllum - Fasting insulin
Intervention
Comparator
Mean Diff. with 95% CI
Weight (%)
Study
N
Mean
SD
N
Mean
SD
Huyen 2010 11 -0.86 6.18 12 3.24 6.84 -4.10 [-9.45, 1.25] 70.57
Huyen 2012 12 17.94 7.06 13 20.02 12.95 -2.08 [-10.36, 6.20] 29.43
Overall -3.51 [-8.00, 0.99]
Heterogeneity: τ² = 0.00, I² = 0.00%, H² = 1.00
Test of θi = θj: Q(1) = 0.16, p = 0.69
Test of θ = 0: z = -1.53, p = 0.13
Favors Gynostemma pentaphyllum as insulin sensitizer
Favors comparator as insulin sensitizer
-10
-5
0
5
Random-effects REML model
